# Supplementary material for: Shared Treg dysregulation underlies psoriasis vulgaris and atopic dermatitis through the PHF19-PRC2 epigenetic axis
Source: Front Immunol. 2026 Aug 20;17:1932788. doi: 10.3389/fimmu.2026.1932788 (PMC13538097; doi:10.3389/fimmu.2026.1932788)
Supplement: Supplementary file 3 [file DataSheet3.zip › Additional files/Additional file 6/04.GINM1.forest.pdf]

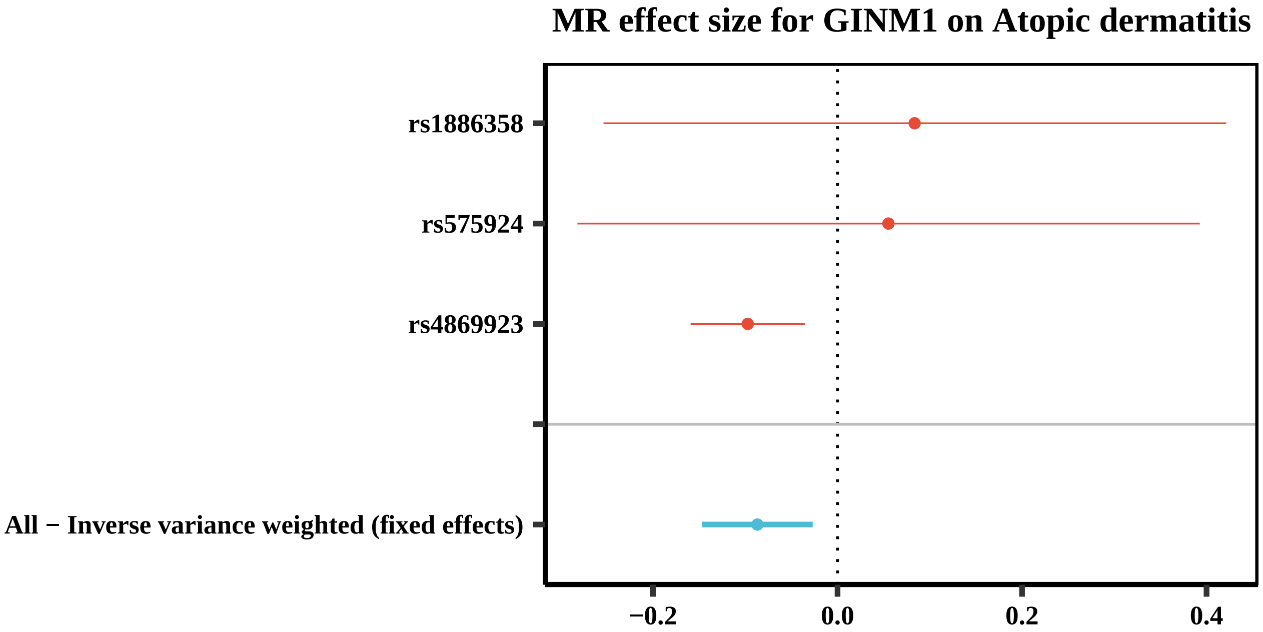
MR effect size for GINM1 on Atopic dermatitis
rs1886358
rs575924
rs4869923
All − Inverse variance weighted (fixed effects)
−0.2
0.0
0.2
0.4
